# Case Report: Abscopal effect in a patient with refractory metastatic hepatocellular carcinoma treated with stereotactic body radiotherapy and PD-1 inhibitor

**DOI:** 10.3389/fimmu.2025.1536689

**Published:** 2025-05-02

**Authors:** Pei Shu, Fang Wang, Xin Wang

**Affiliations:** ^1^ Division of Abdominal Tumor Multimodality Treatment, Cancer Center, West China Hospital, Sichuan University, Chengdu, China; ^2^ Department of Radiation Oncology, Cancer Center, West China Hospital, Sichuan University, Chengdu, China

**Keywords:** hepatocellular carcinoma, immunotherapy, stereotactic body radiotherapy, abscopal effect, HBV

## Abstract

There is currently no established standard treatment for patients with metastatic hepatocellular carcinoma after resistance to tyrosine kinase inhibitors. Given that radiotherapy can reprogram the tumor microenvironment and convert “cold” tumors into “hot” tumors, combining radiotherapy with immunotherapy shows significant potential. In this case, we present a male patient with HBV-related metastatic hepatocellular carcinoma (HCC) accompanied by portal vein tumor thrombosis. The patient achieved abscopal effect with a progression-free survival of 10 months and an overall survival of 21 months with the combination of anti-PD-1 therapy and stereotactic body radiotherapy (SBRT) as third-line treatment. This combination therapy demonstrates relative efficacy and favorability in treating HBV-related advanced HCC.

## Introduction

Hepatocellular carcinoma (HCC) is the sixth leading cause of cancer morbidity and the third major cause of cancer-related death globally, with a death toll of 830,180 in 2020. While a fortunate few are deemed suitable for surgical intervention upon early diagnosis, the majority of cases are detected at advanced stages (1). Targeted therapy has emerged as a standard treatment for advanced HCC with extrahepatic metastases. However, the efficacy of current options remains limited, with sorafenib demonstrating a median time to progression of only 2.8 months (2), and median overall survival (OS) times for sorafenib and lenvatinib standing at 12.3 and 13.6 months, respectively (3). The suboptimal outcomes associated with these therapies are often attributed to the development of drug resistance (4).

Immunotherapy has been recognized as a viable choice for patients with advanced HCC who have failed sorafenib and lenvatinib. However, the response rate of immune checkpoint inhibitors (ICIs) in advanced HCC remains low, at less than 20% (5). Objective response rate of regorafenib combined with sintilimab reached 30% as a second-line treatment, which was superior to regorafenib monotherapy (6). Radiation therapy, particularly stereotactic body radiotherapy (SBRT), not only exerts direct cytotoxic effects on cancer cells but also stimulates a potent antitumor immune response by modulating the tumor microenvironment (7). Encouraging results have been reported by scholars regarding disease control with the combination therapy of radiotherapy (RT) and immunotherapy (8–10). Nevertheless, there is limited research on the abscopal effect of integrating RT and immunotherapy in HCC (11). Here, we present a case of advanced HCC with distal lymph node metastasis that experienced abscopal effect with a prolonged period of disease control and improved survival following treatment with SBRT and nivolumab as third-line therapy.

## Case description

In June 2017, a 45-year-old male initially presented with a mass on the right side of neck. Subsequently, he underwent a biopsy of the enlarged lymph node, and the pathological examination confirmed metastatic carcinoma at a local hospital. On June 19, 2017, the patient was referred to our clinic for additional assessment. An abdominal computed tomography (CT) scan revealed an infiltrative mass in the upper right anterior and left inner regions of the liver, accompanied by multiple metastatic lymph nodes in the hepatogastric, hepatoduodenal, and aortocaval areas. The tumor marker alpha-fetoprotein (AFP) was 948.5 ng/ml (normal range ≤ 8 ng/ml), and the protein induced by the absence of vitamin K or antagonist-II (PIVKA-II) level was 387 mAU/ml (normal range ≤ 32.5 mAU/ml). Additionally, he had chronic hepatitis B virus (HBV) infection with a DNA level of 8.06E+04 IU/ml. he was clinically diagnosed with advanced hepatocellular carcinoma (stage C according to the Barcelona Clinic Liver Cancer criteria), with an Eastern Cooperative Oncology Group (ECOG) performance status of 0 and a Child-Pugh class of A.

**Figure 1:**
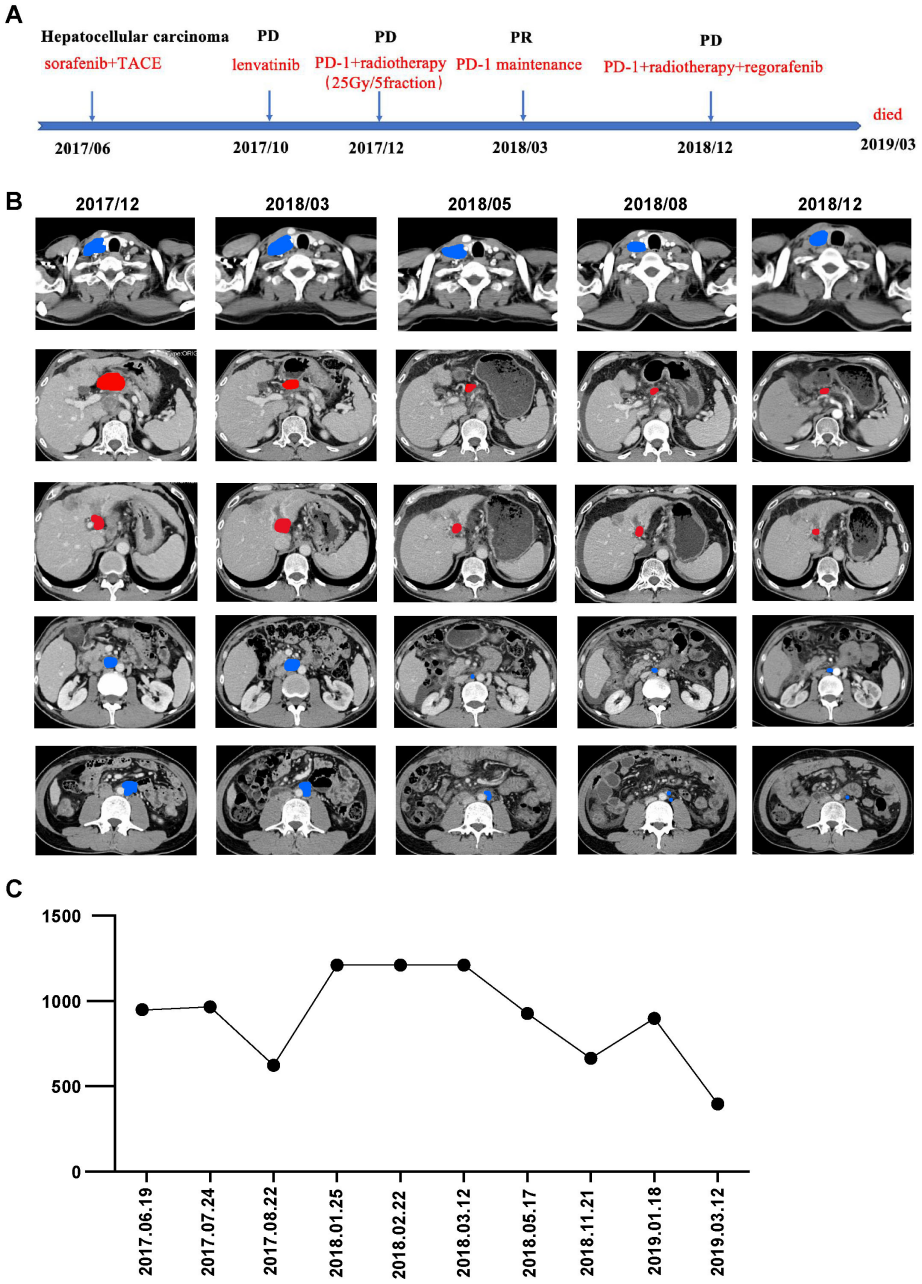
**(A)** Timeline of the whole treatment process for the patient. PD, progressive disease; PR, partial response; SBRT, stereotactic body radiotherapy; PVTT, progressive sagittal portal vein thrombosis. **(B)** CT scans before (2017-12) and after (2018-03,2018-05,2018-08,2018-12) the combination therapy of immunotherapy and SBRT. From top to bottom are right supraclavicular lymph node, hilar lymph node, right para-aortic lymph node, left para-aortic lymph node and PVTT lesions gradually shrunk significantly. Red, irradiated tumor sites; Blue, non-irradiated tumor sites. **(C)** Dynamics of alpha-fetoprotein(AFP)(IU/mL) levels during the entire disease course.

The patient underwent comprehensive therapy as first-line treatment, consisting of local transarterial chemoembolization to the liver lesion and oral sorafenib. Additionally, entecavir 0.5mg was orally prescribed for daily use. After two months, the AFP level dropped to 622.8 ng/ml, and a CT scan showed a stable disease (SD) according to the Response Evaluation Criteria in Solid Tumors (RECIST v1.1). However, enlargement of the abdominal lymph nodes were detected by CT scan, and the AFP level was 926.8 ng/ml after four months, which was assessed as progressive disease (PD). Coincidentally, a phase III trial (REFLECT) comparing lenvatinib with sorafenib in first-line therapy demonstrated non-inferiority objective response rates and progression-free survival (PFS) (12). The RESORCE study, published in 2017, has demonstrated that regofenib can provide survival benefit in patients following progression of sorafenib. However, this drug was not chosen because it was not available in China at the time. Consequently, the patient switched to oral lenvatinib in October 2017. Unfortunately, subsequent CT scans after two months revealed progressive sagittal portal vein thrombosis (PVTT) and enlargement in the hepatogastric lymph nodes. Meanwhile, the serum AFP level increased to more than 1210 ng/ml. The patient was assessed as PD again. A summary of the treatment history is presented in **Figure 1A**.

With informed consent, immunotherapy in combination with SBRT to PVTT and abdominal lymph nodes near the hilum of the liver was determined as third-line therapy. The patient began receiving nivolumab (240mg), an anti-PD-1 antibody, at 2-week intervals in December 2017. We administered 5Gy×5 fractions of radiotherapy, delivered every other day using volumetric modulated arc therapy, following two cycles of nivolumab. Three months after the nivolumab treatment, a follow-up CT scan revealed significant shrinkage of the PVTT and hilar lymph nodes within the irradiation field, along with a continuous decline in AFP levels (**Figures 1B, C**). Surprisingly, there was also notable shrinkage of metastases in the aortocaval areas and right supraclavicular lymph nodes outside of the irradiation field, resulting in a partial remission (PR).

As the regimen showed effectiveness, systemic therapy with nivolumab was continued for one year. The patient tolerated the combination treatment well, experiencing only slight adverse effects such as grade 1 anemia and grade 1 nausea, which were resolved with symptomatic medication. A follow-up CT scan showed metastasis in the anterior mediastinal lymph nodes and lymph nodes behind the vena cava. Subsequently, the patient received systemic therapy with nivolumab and regorafenib, in addition to palliative conventional fractionated radiotherapy for newly enlarged lymph nodes. However, due to complications arising from decompensated cirrhosis, the patient discontinued treatment and unfortunately passed away in March 2019. The OS period reached 21 months, and the timeline of significant clinical events is depicted in **Figure 1**.

## Discussion

In the CheckMate 040 trial, 20% of patients who was treated with nivolumab as second-line treatment experienced an objective response (5). In the phase 2 trial, KEYNOTE-224 study, pembrolizumab obtained response rate of 17% (13). While ICIs have significantly improved efficacy for advanced hepatocellular carcinoma, the issue of drug resistance remains profoundly problematic (14). Response rates of only 15% for single-agent immunotherapy and 30% for two-agent immunotherapy fall far short of clinical needs (15).RT induces immunogenic cell death, remodel the immune microenvironment, overcome immune depletion, and sensitize tumors to immunotherapy (16–19). Specifically, RT stimulates the release of tumor antigens, promotes danger-associated molecular patterns (DAMPs), generates reactive oxygen species (ROS), and increases the expression of major histocompatibility complex I and FAS, leading to anti-tumor activity (20–22). However, radiation therapy also upregulates programmed cell death-Ligand 1, leading to radio resistance, which can be overcome by combining it with ICIs (23). Preclinical studies have shown that combining RT with immunotherapy results in a significant increase in intertumoral T-cell infiltration, tumor regression, and survival compared to RT or immunotherapy alone (24).

SBRT offers the advantage of precisely targeting the lesion while minimizing damage to surrounding healthy tissue, making it more immunogenic compared to conventional fractionated radiotherapy (25). For patients with advanced HCC, particularly those with portal vein tumor thrombosis and inferior vena cava tumor thrombosis, SBRT has demonstrated benefits (26, 27). In a reported case, five patients with unresectable hepatocellular carcinoma who received SBRT and anti-PD-1 antibody showed two complete responses (CR) and three PR (28). However, there is a lack of prospective trial data on the combination of SBRT and immunotherapy. In a phase II clinical trial, patients receiving combination therapy as second-line treatment exhibited a median PFS of 5.8 months and an OS of 14.2 months (29). Additionally, in a propensity score matching analysis comparing SBRT combined with immunotherapy (SBRT-IO) with transarterial chemoembolization (TACE) in patients with locally advanced HCC, the 12-month PFS and 12-month OS were significantly better in the SBRT-IO arm (93.3% vs. 16.7% and 93.8% vs. 31.3%, respectively; P < 0.001) (30).

The presence of underlying HBV infection may influence the selection and effectiveness of treatment strategies. Patients testing positive for HBV exhibited a limited response to sorafenib (31). A study conducted by Pfister D et al. revealed that ICIs therapy had a lower response rate in non-viral HCC compared to viral HCC (32). This finding was corroborated by the results of the IMbrave150 trial, which demonstrated a notable improvement in the prognosis of patients with viral HCC following a combination treatment of atezolizumab and bevacizumab (33). Based on these findings, ICIs therapy may be more suitable for individuals with HBV-associated HCC, while targeted therapy may be more effective for those with non-HBV-related HCC. In the phase 3 KEYNOTE-240 trial, median OS and PFS in patients with advanced HCC, previously treated with sorafenib, was greater for pembrolizumab than for the placebo(OS 13.9 months versus 10.6 months,P=0.0238; PFS 3.0 months versus 2.8 months,P=0.0186) (34). Another phase 3 KEYNOTE394 trial mainly involved Asian patients, with a significantly higher proportion of hepatitis patients than KEYNOTE-240 trial. Median OS were 14.6 months for pembrolizumab and 13 months for placebo, respectively(P=0.018). And median PFS were 2.6 months for pembrolizumab and 2.3 months for placebo (P=0.0032), respectively (35). Therefore, we hypothesize that HBV-associated advanced HCC may possess immunogenic characteristics, potentially enhancing its responsiveness to emerging immunotherapeutic strategies. Attention should be given to the management of HBV-related cirrhosis. In this study, disease progression was observed in patients who discontinued treatment during the later stages due to decompensated cirrhosis. These findings highlight the significance of long-term treatment tolerability and the potential relationship between treatment-related toxicity and hepatic dysfunction, underscoring the necessity for careful assessment and continuous monitoring of liver function and hepatic status in future clinical management.

It has been widely recognized that RT can trigger a systemic immune response, with the abscopal effect being one of the most notable examples. This effect occurs when radiation applied to one site leads to the regression of tumors at distant, non-irradiated sites (36). RT-induced reprogramming of the tumor microenvironment serves as a “game changer”, transforming “cold” tumors, with limited immune cells infiltration, into “hot” tumors, which are characterized by lymphocytic infiltration (37, 38). This transformation creates a favorable condition for the response to immune checkpoint inhibitors (39, 40). Regarding the rationale, the predominant role of combination RT and immunotherapy has been well established (41). We considered this case as an abscopal effect of RT; however, the possibility that it was an immunotherapy effect cannot be ruled out.

Several important questions remain, particularly concerning the optimal RT dose. The immune response induced by RT is dose-dependent, suggesting that the optimal dose should maximize tumor immunity while remaining tolerable for patients. Previous studies indicate that hypofractionated radiotherapy can enhance the coordination between RT and immunotherapy (42, 43). In our study, the dose of 5 Gy × 5 fractions appeared to result in effective disease control. However, there is still limited experience regarding the optimal SBRT dose and segmentation modalities. The study by Dewan et al. demonstrated that a dose of 24 Gy delivered in three fractions (8 Gy × 3 fractions) resulted in superior local tumor control and distant effects compared to two other regimens (20 Gy × 1 fraction or 6 Gy × 5 fractions) (44). Previous studies have indicated that the most common grade 3/4 treatment-related adverse events were elevated transaminases, colitis, or skin reactions, with no occurrences of grade 4 or 5 treatment-related toxicities (7, 28). The timing of ICIs in combination with RT remains a topic of debate. Some studies have provided evidence that administering ICIs and RT concurrently leads to the most significant improvement in OS (45). Moreover, a mathematical model suggests that the maximum response is observed when RT and ICIs are administered simultaneously, and the response diminishes notably with increased time intervals between RT and ICIs (46). Currently, there are several trials exploring the feasibility of SBRT and ICIs, two of which are specific to microvascular invasion (MVI) (NCT04167293, NCT04169399).

A limitation of this case report is the lack of analysis of immunological parameters, which could have strengthened the hypothesis that the observed effects are of immunological origin. Monitoring factors such as immune cells dynamics, as well as longitudinal changes in inflammatory cytokines and growth factors in the patient’s peripheral blood, would have provided valuable insights.

## Conclusion

In this paper, we present a case of HBV-associated advanced HCC with lymph node metastases, which developed an abscopal effect after a combination of immunotherapy and radiotherapy. The factors responsible for inducing the abscopal effect remain unclear and warrant further exploration through additional clinical trials in the future.

## Data Availability

The original contributions presented in the study are included in the article/supplementary material. Further inquiries can be directed to the corresponding author.
